# A hemodialysis cohort study of protocol-based anticoagulation management

**DOI:** 10.1186/s13104-017-2381-7

**Published:** 2017-01-26

**Authors:** S. Lamontagne, Tinzar Basein, Binyue Chang, Lakshmi Mallela

**Affiliations:** 1 Department of Medicine, University of Massachusetts Medical School, Worcester, USA; 2 Division of Nephrology, Berkshire Medical Center, Pittsfield, MA USA; 3 Department of Internal Medicine, Berkshire Medical Center, Pittsfield, MA USA

**Keywords:** Hemodialysis, Warfarin, Anticoagulation

## Abstract

**Background:**

Chronic hemodialysis patients frequently require anticoagulation treatment with warfarin for a variety of co-morbidities. The optimal method for monitoring and dose adjustment of warfarin-based anticoagulation in this population, however, remains unclear. To examine this more closely, we reviewed all hemodialysis patients at a single institution on chronic warfarin therapy for a 10-month period prior to and after the institution of a standardized protocol for warfarin dose adjustment and monitoring. Anticoagulation efficacy was assessed by time within the therapeutic INR range (TTR), and resource utilization was assessed by the number of weekly INR measurements required for monitoring.

**Results:**

We retrospectively analyzed 4481 patient-days of warfarin therapy data (from 25 hemodialysis patients) in the pre-protocol timeframe, and 3308 patient-days of warfarin therapy data (from 21 hemodialysis patients) in the on-protocol timeframe. Time within the therapeutic INR range (TTR) did not improve with institution of the dosing protocol—51.18% using non protocol-based management, and 51.57% using protocol-based management (p 0.73). However, overall resource utilization was reduced with institution of protocolized warfarin monitoring—from 1.71 INR measurements per patient-week pre-protocol, to 1.20 INR measurements per patient-week (p < 0.0001) post-protocol.

**Conclusions:**

In this single-center study, institution of a standardized dosing protocol in a hemodialysis population on chronic warfarin therapy did not improve the rate of on-target anticoagulation, but did result in significantly lower resource utilization. We support protocol-based warfarin management in the hemodialysis population, but future work should examine the rate of on-target anticoagulation typically achieved in this group.

**Electronic supplementary material:**

The online version of this article (doi:10.1186/s13104-017-2381-7) contains supplementary material, which is available to authorized users.

## Background

Anticoagulation therapy with warfarin is commonly prescribed in hemodialysis patients, likely due to the high burden of co-morbidities (atrial fibrillation, thromboembolic disease) necessitating systemic anticoagulation [1]. Although the advent of novel oral anticoagulants may change the preferred pharmacotherapy for anticoagulation in the general population, none of the newly-available anticoagulants are approved for use in End-Stage Renal Disease (ESRD) and warfarin will likely remain the primary oral anticoagulant in this population. Management of warfarin-based anticoagulation in hemodialysis patients is challenging for several reasons. First, there is frequently a shared management between those providers who draw the labs needed to monitor warfarin effect (often done at a patient’s hemodialysis unit) and those who prescribe and dose titrate warfarin (often a primary care or non-nephrology provider). Secondly, given the relative ease and availability of blood sampling in patients on hemodialysis, there may be an “availability bias” towards more frequent lab monitoring and therefore more frequent warfarin dose adjustment in this population.

The optimal means of dose titration for long-term warfarin therapy in the general population has been suggested to include a standardized dose adjustment protocol, rather than individual provider-determined dosing [2]. The optimal means of dose titration in a hemodialysis population (via a dose adjustment protocol or individual provider-determined dosing) remains unclear [3].

In this study, we investigated whether the institution of a standardized protocol to guide lab monitoring and dose adjustment of warfarin therapy would provide better anticoagulation efficacy than non-standardized provider-determined anticoagulation dosing in the hemodialysis population of Berkshire Medical Center (caring for an average of 100 chronic hemodialysis outpatients at any time). At this dialysis center, dialysis providers both obtain labs necessary to monitor chronic warfarin anticoagulation as well as make dose adjustments in warfarin therapy for all patients on chronic anticoagulation.

## Methods

**Fig. 1:**
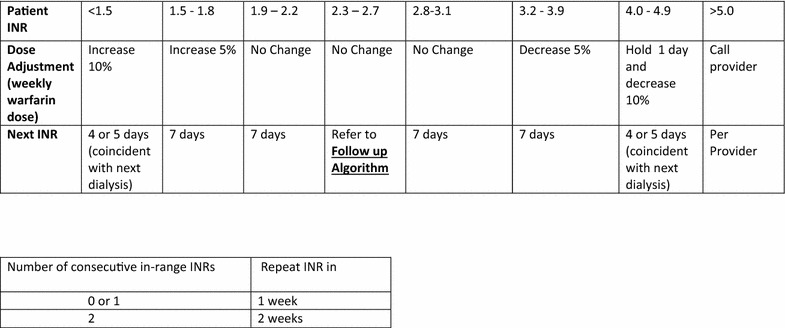
Warfarin monitoring and dose adjustment protocol for Target INR 2.0–3.0 Follow up Algorithm

**Fig. 2:**
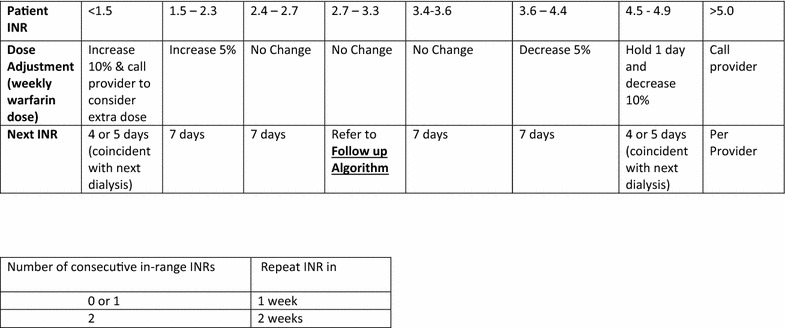
Warfarin monitoring and dose adjustment protocol for Target INR 2.5–3.5 Follow up Algorithm

Under IRB approval, we created a standardized warfarin dosing protocol (agreed upon by nephrology providers) which specified the optimal interval for lab monitoring with INR (International Normalized Ratio) as well as the appropriate warfarin dose adjustment. Two separate protocols were created—one for low-intensity warfarin anticoagulation (Fig. 1, for INR target range 2.0–3.0), and one for high-intensity warfarin anticoagulation (Fig. 2, for INR target range 2.5–3.5). These protocols (Figs. 1, 2) were based on similar institutional protocols used for the general population.

All dialysis nursing staff were educated in the appropriate use of the protocol, including the use of reference tables to assist with easy calculation of any necessary warfarin dose adjustments. Nursing staff was charged with implementation of this protocol, such that the majority of dose adjustments were made without nephrology provider input. However, at extreme ranges of sub-therapeutic or supra-therapeutic anticoagulation, individual provider judgment was the protocol-specified method for determining warfarin dose adjustment and subsequent lab monitoring.

Pre-protocol (baseline) anticoagulation efficacy data was collected for all hemodialysis patients on chronic warfarin therapy at our institution for the 10-month period from January, 2013 to October, 2013. Per institutional IRB guidance, informed consent from individual participants was not required or obtained as only de-identified laboratory data was collected and analyzed. Post-protocol (intervention) anticoagulation efficacy data was collected for the 10-month period of December, 2013–September, 2014. Our primary outcome was the efficacy of anticoagulation therapy, as defined by time within the therapeutic INR range (TTR), and was calculated by the Rosendaal method [4]. A secondary outcome was resource utilization, as defined by the number of lab assessments (INR values), required for anticoagulation management. Statistical significance for the two major outcomes (TTR and frequency of INR monitoring) was calculated using a two sample percent defective analysis.

As the intention of this study was to assess the efficacy of a dosing protocol in *chronic* anticoagulation management—we excluded data from any time period for which there was an extended gap (defined as over 14 days) in outpatient warfarin monitoring (due to intercurrent hospitalization, missed outpatient dialysis sessions, etc.…). It was assumed that during these extended time gaps that warfarin therapy could have been stopped and restarted, and the time limit was chosen as 14 days as this was the longest monitoring interval specified in our warfarin management protocol. In situations where interruptions in outpatient warfarin monitoring were present, the pre-interruption and post-interruption time periods were analyzed as separate anticoagulation spans. All INR measurements were drawn at the start of a dialysis session.

## Results

We collected 4481 patient-days of warfarin therapy data (from 25 hemodialysis patients) in the pre-protocol timeframe, and 3308 patient-days of warfarin therapy data (from 21 hemodialysis patients) in the post-protocol timeframe. Patient characteristics for those hemodialysis patients included in the pre and post-protocol analyses are summarized in Table 1.

**Table 1:** Patient characteristics for pre-protocol and post-protocol timespan

	Pre-protocol	Post-protocol
n	25	21
% Male/female	64/36	57/43
Mean age (as of 11/1/2013) ± standard deviation	70 ± 14	61 ± 16
*ESRD diagnosis*		
Diabetic nephropathy	9	7
Hypertension	4	3
Polycystic kidney disease	2	2
Glomerulonephritis	2	4
Renal artery stenosis	2	1
Other	6	4
*Indication for warfarin*		
Atrial fibrillation	17	8
DVT/PE	2	5
Mechanical cardiac valve	1	2
Other	5	6
*INR target range*		
2–3	20	17
2.5–3.5	4	3
Other	1	1

Our primary endpoint, the efficacy of anticoagulation as defined by TTR, was 51.18% using non-protocolized management, and 51.57% using protocol-based management (p 0.73, Table 2). Our secondary endpoint, the number of INR measurements utilized, was 1.71 per patient-week using non-protocolized management, and 1.20 per patient-week using protocol-based management (p < 0.0001, Table 2).

**Table 2:** Primary and secondary outcome data

	Non-protocol management	Protocol-based management	p value
Time in therapeutic range (TTR)— %	51.18	51.57	0.73
INR measurements (per patient-week)	1.71	1.20	<0.0001

## Discussion

Chronic warfarin-based anticoagulation is commonly prescribed in the hemodialysis population within the United States and is likely to remain a common treatment in this population in the foreseeable future. In this single-center hemodialysis population, we achieved a relatively low rate of on-target anticoagulation (51%) by instituting a warfarin dosing protocol, and there was no statistically-significant improvement in overall anticoagulation efficacy using a protocol-based dosing method versus a non-standardized provider-based dosing method.

There are several possible explanations for the lack of improved anticoagulation outcomes after the implementation of a protocol-based dosing method as was observed in our study. First, it is possible that our primary outcome measure (time within the therapeutic INR range, TTR) was an inaccurate means to assess the true rate of on-target anticoagulation. This appears unlikely given that measurement of on-target anticoagulation by another statistical methodology—simply the percentage of in-range INR measurements—yields very similar results (50.5% on-target anticoagulation using non-protocolized management, versus 48.4% on-target anticoagulation in the protocol-based management strategy). Second, although nursing adherence to the warfarin protocol was mandated (by dialysis unit policy) throughout the study period, dialysis provider adherence was not. The majority of warfarin dose adjustments were made by nursing staff, but individual provider input was specified per protocol in the extremes of subtherapeutic or supratherapeutic anticoagulation, and decision-making in these “non-standardized” situations may have skewed the overall benefit of protocol-based management. Third, the providers managing warfarin dose adjustments in the pre-protocol timeframe have substantial experience in doing so, and prior research has shown that provider experience in managing warfarin dose adjustments can attenuate the benefit of implementation of a dose adjustment protocol [2].

This study did show a substantial benefit of protocol-based warfarin management in terms of resource utilization, with 30% fewer INR measurements needed to achieve a similar anticoagulation efficacy in the protocol-based management strategy. Further, the use of provider time required for dose adjustment was far less utilizing a protocol rather than reviewing a patient’s prior warfarin dose schedule and determining a new dose in each instance (Additional file 1).

To our knowledge, only one prior study has similarly examined the effect of institution of a warfarin dosing protocol in a hemodialysis population, and this found similar outcomes—no improvement in the rate of therapeutic INR with use of protocol-based dosing, but a reduction in INR measurement utilization with use of a protocol [3] (Additional file 2).

Our study was underpowered to examine the clinical benefit (incidence of thromboembolic stroke, recurrent DVT or PE, etc.…) or clinical harm (bleeding complications) of these two warfarin dosing strategies, however TTR has been validated as a surrogate marker for clinical outcomes in the non-dialysis population [5]. Specifically, prior research has shown that the clinical benefit of anticoagulation in atrial fibrillation (a very common indication for warfarin therapy in the ESRD population) depends very much on achievement of the target INR—with substantially greater benefit in centers achieving TTR >65% versus those who achieve <65% [6]. If the on-target anticoagulation rate in our study is reflective of the typical TTR in the hemodialysis patient population at large it could, in part, explain the large degree of uncertainty as to the overall benefit of warfarin anticoagulation (especially for stroke prevention in atrial fibrillation) in the dialysis population, as compared to the general population [7, 8].

## Conclusion

This study showed equivalent anticoagulation efficacy but significantly lower resource utilization by implementing a warfarin dose adjustment protocol in a hemodialysis population. We support the use of warfarin dosing protocols for all providers responsible for managing chronic anticoagulation in an ESRD population.

## Additional files

**Additional file 1.** Pre-protocol final data—Includes a summation of individual patient demographic data, as well as rates of on-target anticoagulation and resource utilization (number of lab assessments) for the non-protocol-based anticoagulation strategy.

**Additional file 2.** Post-protocol final data—includes a summation of individual patient demographic data, as well as rates of on-target anticoagulation and resource utilization (number of lab assessments) for the protocol-based anticoagulation strategy.

## Abbreviations

INR: international normalized ratio
TTR: time within the therapeutic INR range
ESRD: end-stage renal disease
